# Optimizing drug discovery for snakebite envenoming via a high-throughput phospholipase A2 screening platform

**DOI:** 10.3389/fphar.2023.1331224

**Published:** 2024-01-11

**Authors:** Laura-Oana Albulescu, Adam Westhorpe, Rachel H. Clare, Christopher M. Woodley, Nivya James, Jeroen Kool, Neil G. Berry, Paul M. O’Neill, Nicholas R. Casewell

**Affiliations:** ^1^ Centre for Snakebite Research and Interventions, Department of Tropical Disease Biology, Liverpool School of Tropical Medicine, Liverpool, United Kingdom; ^2^ Centre for Drugs and Diagnostics, Department of Tropical Disease Biology, Liverpool School of Tropical Medicine, Liverpool, United Kingdom; ^3^ Department of Chemistry, University of Liverpool, Liverpool, United Kingdom; ^4^ Division of BioAnalytical Chemistry, Department of Chemistry and Pharmaceutical Sciences, Faculty of Science, Amsterdam Institute of Molecular and Life Sciences, Vrije Universiteit Amsterdam, Amsterdam, Netherlands

**Keywords:** high-throughput screening, drug discovery, toxin inhibitors, snake venom, snakebite envenoming, neglected tropical disease

## Abstract

Snakebite envenoming is a neglected tropical disease that causes as many as 1.8 million envenomings and 140,000 deaths annually. To address treatment limitations that exist with current antivenoms, the search for small molecule drug-based inhibitors that can be administered as early interventions has recently gained traction. Snake venoms are complex mixtures of proteins, peptides and small molecules and their composition varies substantially between and within snake species. The phospholipases A2 (PLA_2_) are one of the main pathogenic toxin classes found in medically important viper and elapid snake venoms, yet varespladib, a drug originally developed for the treatment of acute coronary syndrome, remains the only PLA_2_ inhibitor shown to effectively neutralise venom toxicity *in vitro* and *in vivo*, resulting in an extremely limited drug portfolio. Here, we describe a high-throughput drug screen to identify novel PLA_2_ inhibitors for repurposing as snakebite treatments. We present method optimisation of a 384-well plate, colorimetric, high-throughput screening assay that allowed for a throughput of ∼2,800 drugs per day, and report on the screening of a ∼3,500 post-phase I repurposed drug library against the venom of the Russell’s viper, *Daboia russelii*. We further explore the broad-spectrum inhibitory potential and efficacy of the resulting top hits against a range of medically important snake venoms and demonstrate the utility of our method in determining drug EC_50_s. Collectively, our findings support the future application of this method to fully explore the chemical space to discover novel PLA_2_-inhibiting drugs of value for preventing severe pathology caused by snakebite envenoming.

## Introduction

Snakebite envenoming is a neglected tropical disease (NTD) that results in ∼140,000 deaths and 400,000 disabilities globally each year (Gutiérrez et al., 2017). It disproportionately affects the low and middle income countries (LMIC) of Asia, Africa, the Americas and Oceania, and predominantly impacts isolated rural communities (Harrison et al., 2009) for whom transportation to a clinic to receive treatment can take >6 h (Sharma et al., 2004; Ogunfowokan, 2012; Longbottom et al., 2018; Mise et al., 2018). The only approved treatment for snakebite is antivenom - animal derived, polyclonal antibody-based products generated via immunisation of equines or ovines. Despite saving thousands of lives annually, antivenom comes with several limitations, including risk of severe adverse reactions such as anaphylaxis (de Silva et al., 2016), high cost, lack of availability in many remote areas, and reliance on the cold chain (Williams et al., 2011). Because of its requirement for intravenous delivery, coupled with the need to manage potentially life-threatening adverse reactions, antivenom can only be administered under medical supervision in a clinical environment, which causes treatment delays and poor patient outcomes. Finally, antivenom is highly species-specific due to snake venom variation, meaning that an antivenom will typically only be efficacious against those venoms included in the immunising mixture (Chippaux et al., 1991; Gutiérrez et al., 2017).

The diversity of snake venoms, each of which can consist of a variable mixture of over >200 peptide and/or protein components (Casewell et al., 2013), also complicates the clinical picture of envenoming. Medically important venomous snakes fall into two main families: Viperidae (vipers) whose bites are typically characterised by causing haemotoxic and/or tissue destructive effects, and Elapidae (elapids) whose venoms are typically neurotoxic (Gutiérrez et al., 2017). This divergence in pathology is driven by differences in the main toxin classes present, with viper venoms often dominated by enzymatic toxins such as snake venom metalloproteinases (SVMPs), group II phospholipase A_2_ (PLA_2_), and snake venom serine proteases (SVSPs), whereas non-enzymatic three-finger toxins (3FTxs) and group I PLA_2_s are often the most abundant in elapid venoms (Tasoulis and Isbister, 2017).

Since PLA_2_s are major contributors to the snakebite pathology caused by most venomous snakes, understanding their mechanisms of action can help us devise new treatments to mitigate their pathological effects. Enzymatic PLA_2_s hydrolyse the sn-2 ester bonds of cell membrane phospholipids, contain a catalytic Asp49 residue and utilise Ca^2+^ as a cofactor. In contrast, non-enzymatic PLA_2_s are Ca-independent and lack a negative charge at position 49, with aspartic acid replaced by either a positive (typically lysine) or non-charged amino acid (Kini, 2003; Gutiérrez and Lomonte, 2013). Anticoagulant enzymatic PLA_2_s are monomeric and have been described in both viper {RVV-PFIIc from *Daboia russelii* (Chakraborty et al., 2002) and elapid [e.g., *Naja nigricollis* (Kerns et al., 1999; Kini, 2005)]} venoms. On the other hand, multimeric (including homo- or hetero-dimeric) PLA_2_s are present in various venoms and can include both enzymatic and non-enzymatic PLA_2_ subunits, or can be formed of complexes with other toxins which facilitate targeting of the PLA_2_s to their site of action. For example, *Oxyuranus scutellatus* venom contains the trimeric catalytically active (Asp49) PLA_2_ taipoxin (Fohlman et al., 1976; Cendron et al., 2012), while members of the pitviper genus *Crotalus* have heterodimeric PLA_2_s which either have [e.g., crotoxin (Sampaio et al., 2010)] or lack (e.g., CoaTX-II (Almeida et al., 2016)) enzymatic activity. Additionally, β-bungarotoxin from the elapid *Bungarus multicinctus* consists of an enzymatic PLA_2_ and a Kunitz-type peptide heterodimer and exerts presynaptic neurotoxicity (Kwong et al., 1995). Intriguingly, enzymatic and non-enzymatic PLA_2_s can also coexist in the same venom. One such example is the venom of the pitviper *Bothrops asper* (terciopelo) which contains two types of monomeric myotoxins: the enzymatic myotoxin-I and the non-enzymatic myotoxin II, both of which destabilise the sarcolemma before being internalised in the cell nucleus (Vargas-Valerio et al., 2021). Given this diverse range of pathological effects, and the near ubiquity of PLA_2_ toxins across the variable venoms of medically important snakes (Tasoulis and Isbister, 2017), broad-spectrum neutralisation of the toxic effects of PLA_2_s would represent a major step in the treatment of snakebite envenoming.

The concept of broadly targeting and neutralising a whole venom toxin class, rather than focusing on species-specific treatments, has recently gained traction in the snakebite community (Bulfone et al., 2018; Clare et al., 2021; Gutiérrez et al., 2021). Small molecule toxin inhibitors capable of neutralising SVMP or PLA_2_ toxins (Rucavado et al., 2004; Lewin et al., 2016; Arias et al., 2017; Lewin et al., 2018a; Lewin et al., 2018b; Bryan-Quirós et al., 2019; Albulescu et al., 2020a; Albulescu et al., 2020b; Menzies et al., 2022), and which can be formulated as oral drugs, have been proposed as rapid interventions to be given to snakebite patients in the field soon after a snakebite. Such treatments could possess several advantages over conventional antivenoms, such as their potential for cross-species neutralisation through generic inhibition of the active site of a class of toxins, improved safety profiles, reduced cost over antibodies, and potential ease of administration (Clare et al., 2021). The efficacy of such drugs has been investigated in the context of both systemic (Rucavado et al., 2004; Arias et al., 2017; Lewin et al., 2018a; Lewin et al., 2018b; Albulescu et al., 2020a; Albulescu et al., 2020b) and local (Bryan-Quirós et al., 2019; Albulescu et al., 2020a; Hall et al., 2023) envenoming in *in vivo* preclinical models, and in the context of both single drug treatments and combinations of inhibitors targeting different toxin families. Among these, varespladib and its orally bioavailable analogue methyl varespladib have been shown to be highly effective in neutralizing the toxicity of both several viper and elapid venoms *in vitro* and *in vivo* (Lewin et al., 2016; Lewin et al., 2018a; Lewin et al., 2018b). Importantly, varespladib appears to target both enzymatic and non-enzymatic PLA_2_s and leads to a conformational change upon binding which renders the toxin unable to access and act upon its substrate (Salvador et al., 2019). Although varespladib was originally developed for the treatment of acute coronary syndrome (Chiara Ricci, 2015), it has recently progressed into human clinical trials (phase II) in India and United States for snakebite indication, in a drug repurposing approach (Lewin et al., 2022). While this is a highly promising development, the snakebite drug portfolio remains limited, with no robust backup PLA_2_-inhibiting molecules in place to offset the risk of attrition or failure during clinical development. This is predominately because no concerted drug discovery screening efforts to identify novel PLA_2_ inhibiting compounds have been described to date.

Here, we present the first high-throughput screen (HTS) for novel inhibitors capable of neutralising the PLA_2_-activity of snake venoms. We describe the methodology and optimisation steps leading to the validation of a 384-well plate colorimetric assay capable of screening ∼2,800 drugs/day, and report our findings from the primary screening of a model ∼3,500 compound, post-phase I, commercial drug library. To account for the potential influence of venom variation and varying inhibitory drug potencies against distinct PLA_2_ isoforms, we further explored our hits for their broad-spectrum efficacy against a panel of diverse snake venoms. We hope that the described methodology will prove valuable to the snakebite community in its search for novel broad-spectrum small molecule inhibitors directed against venom PLA_2_ toxins.

## Methods

### Venoms

Venoms were sourced from either wild-caught specimens maintained in, or donated historical venom samples stored in, the herpetarium of the Liverpool School of Tropical Medicine. The venom pools selected encompassed vipers and elapids from diverse geographical localities and were from: *D. russelii* (Sri Lanka), *B. arietans* (Nigeria), *N. nigricollis* (Tanzania), *C. atrox* (United States)*, N. naja* (captive bred) and *C. durissus terrificus* (Brazil). Crude venoms were lyophilized and stored at 4 °C to ensure long term stability. Prior to use, venoms were resuspended to 10 mg/mL in PBS (pH 7.4, Cat no: 10010023, Gibco) and then further diluted to 1 mg/mL stock solutions in PBS for the described experiments.

### PLA_2_ assay optimisation

The Abcam sPLA_2_ assay (Cat No: ab 133089, Abcam) functions by measuring the rate of hydrolysis of the sn-2 bond of a phosphatidylcholine analogue, diheptanoyl-thio-phosphatidylcholine, by secretory PLA_2_s (sPLA_2_s). Once hydrolysed, this substrate yields a free thiol group that interacts with 5,5′-dithio-bis-(2-nitrobenzoic acid) (DTNB), which is then converted into 2-nitro-5-thiobenzoic acid, causing a change in colour which is measurable at 405 nm. This conversion is measured kinetically and occurs at a linear rate until the substrate is exhausted, at which point the absorbance plateaus. Appropriate PLA_2_ concentrations are determined to fit within the linear range of the assay and a bee venom positive control is included with the kit. The assay is designed for 96-well plates and is quite costly at GBP£440/96 tests at the time of writing. We wanted to adapt and miniaturise this methodology for high-throughput screening of PLA_2_ activity and inhibition in 384-well plates to decrease cost and increase output, without compromising assay robustness.

To this end we miniaturised the assay 5-fold to a working volume of 45 μL, decreased the stock concentration of the substrate from 1.66 to 1.33 mM, and modified the reaction volumes to enable the use of robotics. The final reaction consisted of 10 µL of snake venom diluted in assay buffer (25 mM Tris-HCl, pH 7.5, 10 mM CaCl_2_, 100 mM KCl, 0.3 mM Triton X-100), 5 µL of 4 mM DTNB in 0.4 M Tris-HCl, pH 8.0 and 30 µL of 1.33 mM substrate stock in assay buffer. The final concentrations of DTNB and that of the substrate in the assay were 0.44 and 0.88 mM, respectively.

To determine PLA_2_ inhibition, 0.5 µL of 1 mM drugs in DMSO (≥99.7%, Cat No: D2650, Sigma) were printed onto 384-well plates using a VIAFLO384 384-channel electronic pipette (Integra). Then, 10 µL of snake venom at a concentration appropriate for the venom being screened was added to the plates using the VIAFLO384 and the plates were sealed with adhesive plate seals (Greiner BioOne), spun down and incubated for 25 min at 37°C. To prevent the degradation of any potential photolabile compounds, the plates were wrapped in aluminium foil prior to the incubation step. The plates were then taken out of the incubator and left to acclimatise at room temperature for 5 min. Five microliters of 4 mM DTNB and 30 µL of 1.33 mM substrate were then added sequentially to the plates using the VIAFLO384, after which the plates were immediately read kinetically on a CLARIOstar plate reader (BMG Labtech) at 405 nm for 15 min (settings for a full 384-well plate were 11 flashes, 161 s cycle time).

### Range finding for venoms

To determine working ranges for the various PLA_2_-rich venoms, serial dilutions were performed for each venom. Initially, for venoms such as *D. russelii* and *N. nigricollis* a large concentration interval was tested ranging from 200 pg to 2 μg, but it was quickly determined that the 1–100 ng interval is usually sufficient to identify an appropriate venom activity that falls within the linear range of the assay. Therefore, for other venoms used for breadth of inhibition tests a smaller concentration window was used.

### Assay validation

Prior to conducting drug library screens, it was important to assess whether any drift or edge effects occurred in our setup. To this end, interleaved plates containing DMSO, assay buffer, 10 µM varespladib (≥98%, Cat no: SML1100, Sigma) and 10 µM marimastat [a matrix metalloproteinase (MMP) inhibitor previously used to inhibit snake venom metalloproteinase venom toxins *in vitro* (Albulescu et al., 2020b); ≥99%, Cat No: M2699, Sigma] were set up. These consisted of full rows of these compounds in a repeating pattern (e.g., DMSO was in rows A, E, I, M, buffer was in rows B, F, J, N, etc.) on a 384-well plate. The assay was conducted as previously described using 20 ng/well of *D. russelii* venom across the plate. This was repeated three times on different days. To determine PLA_2_ activity under each of these conditions, the slope of each reaction was calculated by dividing the difference in absorbance at 405 nm between the first two time points in the linear range (t_n_–t_0_) by time (in min) where t_n_ was 2 min 41 s in this case. These values were then inputted into the following equation:

$$A\;\left(\frac{µ\text{mol}}{\min\times \text{mL}}\right)=\frac{\text{slope}\times 0.045}{\left(4.6\times 0.01\right)}$$

where A represents venom activity, 0.045 is a factor representing the total volume of the assay (45 µL), 4.6 is the extinction coefficient of DTNB in 384-well plates, and 0.01 represents the sample size of 10 µL. This activity is then converted into specific activity by dividing the overall activity to the amount of venom in the reaction in micrograms.

To assess the quality of the assay, we calculated Z prime values across the plate by using the following formula:

$$Z^{\prime }=1-\frac{3\left(\sigma \;\left(pos\right)-\;\sigma \;\left(neg\right)\right)}{\left|µ\;\left(pos\right)-\;µ\;\left(neg\right)\right|}$$

where σ and µ represent the standard deviations and means of the positive (varespladib containing wells) and negative controls (DMSO only containing wells), respectively. Acceptable assay Z′s are ≥ 0.4, with increased values up to 1 representing highly robust datasets. Plate means and coefficients of variation for the positive and negative controls were also calculated.

### Primary screening of the drug library

Following assay validation in 384-well format, a bespoke repurposed drug library of 3,547 compounds was purchased from MedChemExpress. This library (a combination of the HY-L035 and HY-L026 libraries with overlaps removed) was curated by MedChemExpress to contain a diverse chemical panel of post-phase I and approved drugs that have completed preclinical and clinical studies for a wide range of diseases. All drugs have well-characterised bioactivity, safety, and bioavailability properties, making them suitable for drug repurposing. Once in house this library was formatted as 11 × 384-well stock plates containing drugs at a concentration of 1 mM in DMSO. These were screened against 2 ng/μL (20 ng/well) of *D. russelii* (Sri Lanka) snake venom, as determined earlier. In addition to the blinded compounds on the plates, control compounds were added to confirm that the assay had performed appropriately. These controls included 10 µM varespladib as a positive control (100% inhibition) and vehicle (DMSO) as a negative control (0% inhibition). Assay-ready plates (ARPs) were made by transferring 0.5 µL from each well of the stock plates onto new 384-well plates, so that the layout of the ARP matched that of the stock plate. Venom was added at 10 µL per well (20 ng venom/well) across the plate, except for column 24 which received 10 µL of assay buffer. These latter wells containing control drugs and assay buffer served as the blank. The plates were incubated for 25 min at 37°C, then left to reach room temperature for 5 min. Next, 5 µL of DTNB, followed by 30 µL of substrate were added sequentially to the wells, and the plate was immediately read kinetically on a CLARIOstar multiwell plate reader at 405 nm (temperature set to 25°C, 15 min total read time, 161 s/read cycle). The data were analysed as above by calculating the slopes of all reactions on the plate. The average of the slopes of the blanks was then subtracted from each of these values, and specific activity calculated as described and expressed as µmol/min/mL/µg of venom.

To determine percentage inhibition relative to the positive and negative controls we applied the following formula:

$$\%\;inhibition=\left(\;\frac{A-\;µ\;\left(pos\right)}{µ\;\left(neg\right)-\;µ\;\left(pos\right)}\right)\times 100$$

where A represents specific activity as determined for each sample, and µ (pos) and µ (neg) represent the means of the specific activity in the positive and negative controls, respectively.

Compounds that demonstrated inhibitory PLA_2_ activity of at least 60% compared to varespladib and venom alone were progressed to allow retesting for confirmation of their activity.

### Rescreening hits from the drug library

Thirty-six compounds from the MedChemExpress library were identified as hits in the primary screen (9 strong at >80% and 27 mediocre between 60% and 80% inhibition of venom PLA_2_ activity). However, considering the primary screen was run in singleton, we wanted to confirm whether 60% inhibition was an appropriate threshold to allow us to identify true positives. These compounds were thus selected and transferred onto one plate for retesting together against *D. russelii* venom (20 ng/well). These experiments were conducted and analysed as above, with *n* = 4 repeats per compound.

### Retesting of top hits

To further confirm our top hits and assess breadth of inhibition across venoms, the following compounds were ordered in from commercial sources: (*R*)-(-)-Gossypol acetic acid (≥98%, Cat No: HY-15464A, MedChemExpress), DL-Borneol (Cat No: 23468, Cayman Chemical), punicalagin (Cat No: 13069, Cayman Chemical), quercetin dihydrate (≥96%, Cat No: HY-N0146, MedChemExpress) and prasugrel hydrochloride (≥98%, Cat No: HY-15284A, MedChemExpress). Drugs were made into 1 mM working stocks in DMSO, after which they were tested at a final concentration of 10 µM against a variety of PLA_2_-rich venoms (*D. russelli*, *N. nigricollis*, *N. naja*, *B. arietans*, *C. atrox* and *C. durissus terrificus*. The amount of venom used in each case has been described above in the “*Range finding for venoms*” section.

### EC_50_ testing

To further explore the capabilities of the assay, EC_50_ curves were generated for drug hits that displayed the best broad-spectrum venom inhibition (gossypol, punicalagin and varespladib) against three venoms: *D. russelii*, *N. nigricollis* and *C. durissus terrificus*. Drugs were plated as before, starting at 10 µM followed by 2-fold dilutions down to 5 pM, with each dilution series plated in quadruplicate. The data were analysed and expressed as described above and the percentage PLA_2_ inhibition values were used to generate EC_50_s in Graphpad Prism 9 using a nonlinear regression to fit the dose-response inhibition curve.

### Computational chemistry

The HTS library was screened for common Pan-Assay INterference compoundS (PAINS) substructures (Baell and Holloway, 2010) using the in-built PAINS filter in the RDKIT cheminformatics package version 2022.03.5 as implemented in Python (Landrum et al., 2020). Tree Manifold Approximation Projection (TMAP) visualisation was performed using the TMAP python module version 1.0.6 (Probst and Reymond, 2020). The DataWarrior open source program was used to calculate the Ligand efficiency metrics (Sander et al., 2015). LogP and heavy atom counts were calculated using StarDrop™ ADME QSAR module (Version 7.3, Optibrium, Ltd., Cambridge, United Kingdom).

## Results

### Optimising a commercial sPLA_2_ assay for HTS screening of drug libraries

**FIGURE 1:**
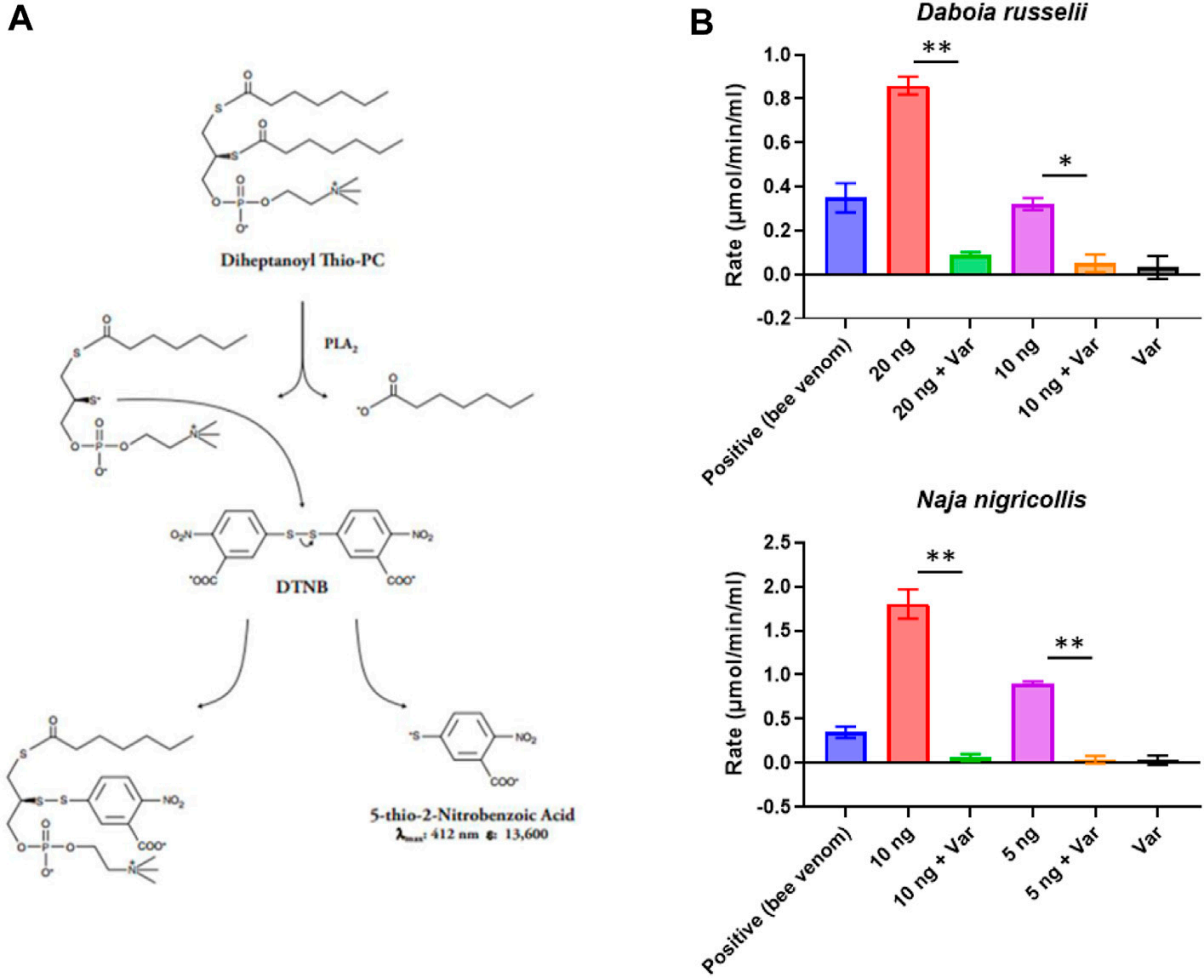
Assay setup and miniaturisation. **(A)** Assay reaction principle. **(B)** Examples of venoms optimised for work under HTS conditions. Various venom concentrations were explored to ensure they were within the linear range of the assay (20, 10 or 5 ng shown here), and then inhibition of PLA_2_ activity by 10 µM varespladib (var) was tested. Data shown represents the reaction rate, with bars showing the mean of duplicate readings and error bars representing standard error. Significance is displayed above the graphs with *p*-values *p* < 0.05 (**) and *p* < 0.5 (*) shown.

To best utilise commercial drug libraries as a source for novel PLA_2_ inhibitors, we first needed to identify and optimise a screening method that would allow us to fully explore the chemical space. To this end, we took advantage of the commercially available Abcam secretory PLA_2_ kit (ab133089), which we miniaturised and adapted for use in 384-well format using robotics (see Methods, Figure 1A). This resulted in a 5-fold scale-down of the assay volume and decreased substrate concentrations to allow increased sample throughput while reducing the cost. Using the kit-supplied bee venom as a positive control, we determined working concentration ranges for a representative viper (*D. russelii*) and elapid (*N. nigricollis*) snake venom, with 10-fold dilutions ranging from 200 pg to 2 µg of venom tested. Our findings demonstrated that 20 ng of *D. russelli* and 5 ng of *N. nigricollis* venom were sufficient to display at least equivalent activity to the positive bee venom control, while still falling within the linear range of the assay (Supplementary Figure S1). We next demonstrated that 10 µM of the gold standard PLA_2_ inhibitor, varespladib, inhibited venom activity to baseline readings for both viper and elapid venoms (Figure 1B).

**FIGURE 2:**
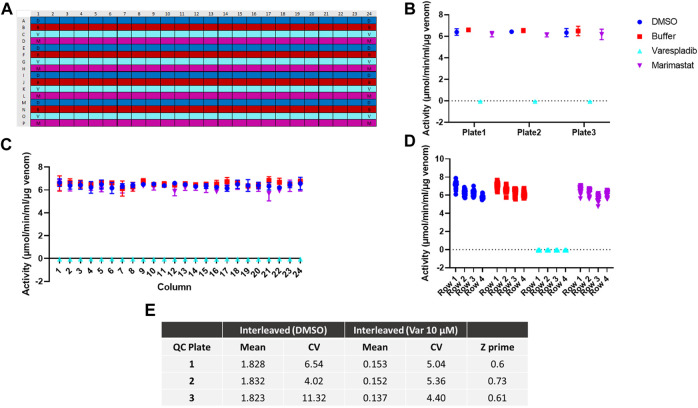
Assay optimisation for high throughput screening. **(A)** Plate map showing interleaved controls across the 384-well plate. D - DMSO (dark blue), B - buffer (red), V - varespladib (light blue), M - marimastat (magenta). **(B)** Signal variability across runs (*n* = 3) expressed as the average activity across each 384-well plate with standard deviation (SD) represented by error bars. **(C)** Signal consistency (*n* = 3) expressed as the average activity for each column across all 384-well plates with corresponding SD shown by error bars. No drift is observed from column 1 to 24. **(D)** The signal variability for each row containing the same control (DMSO, buffer, 10 µM varespladib or 10 µM marimastat). The average of all 24-data points/row across 3 plates is displayed for each condition—e.g., for DMSO/venom only, rows 1–4 indicate rows A, E, I and M. A minimal decrease in activity is noted from A to M, but this still allows for a significant window of detection between the positive and negative controls. **(E)** Quality control statistics for each interleaved plate tested including mean values, coefficients of variation (CVs) of controls and Z′ values.

To further test the applicability of our adapted assay for large scale screening we next investigated assay reproducibility across the 384-well plate and replicability between different batches of 384-well plates. To determine intra-plate and inter-plate variability, we ran three identical interleaved plates containing *D. russelii* venom on different days. The plate setup (Figure 2A) accounted for all drugs being dissolved in DMSO; as such the DMSO-only control is a proxy for venom alone (negative control), whereas the varespladib control represents a positive control (100% inhibition). Marimastat, a peptidomimetic inhibitor of a different class of venom toxins (the snake venom metalloproteinases), and assay buffer, both of which are not expected to neutralise PLA_2_ activity, were also included in the panel as additional controls (marimastat at a concentration of 10 µM). The signals across the plate after blank subtraction were expressed as activity (µmol/min/mL/µg venom) and are displayed in Figures 2B–D. The venom only controls (labelled “DMSO”) and the two controls, which do not inhibit PLA_2_ activity, displayed values of 6–8 μmol/min/mL/µg venom, which are venom-dependent. When PLA_2_ activity was inhibited with varespladib this dropped sharply, allowing us to establish a baseline (100% inhibition). No differences were noted between the average signal values on each of the three plates, suggesting good reproducibility of the method (Figure 2B). Similarly, when variability was assessed on a column or row basis, we noted no drift across columns (1–24) and only a minimal change in activity from the top to bottom rows – a reflection of the time it takes the instrument to read the plate in real time (Figures 2C, D). Our data suggest that under these conditions we can obtain a reliable assay window between our positive and negative controls in which to measure PLA_2_ inhibition by various drugs. The full plate by plate data is provided in Supplementary Figure S2. We calculated resulting Z-primes, which represents a measure of assay quality (Zhang et al., 1999), where the threshold routinely set for such assays is indicated by a Z′ > 0.5. Values from each of our three runs were ≥0.6, thereby confirming the robustness of our method (Figure 2E).

### Screening a commercial drug library for novel PLA_2_ inhibitors

**FIGURE 3:**
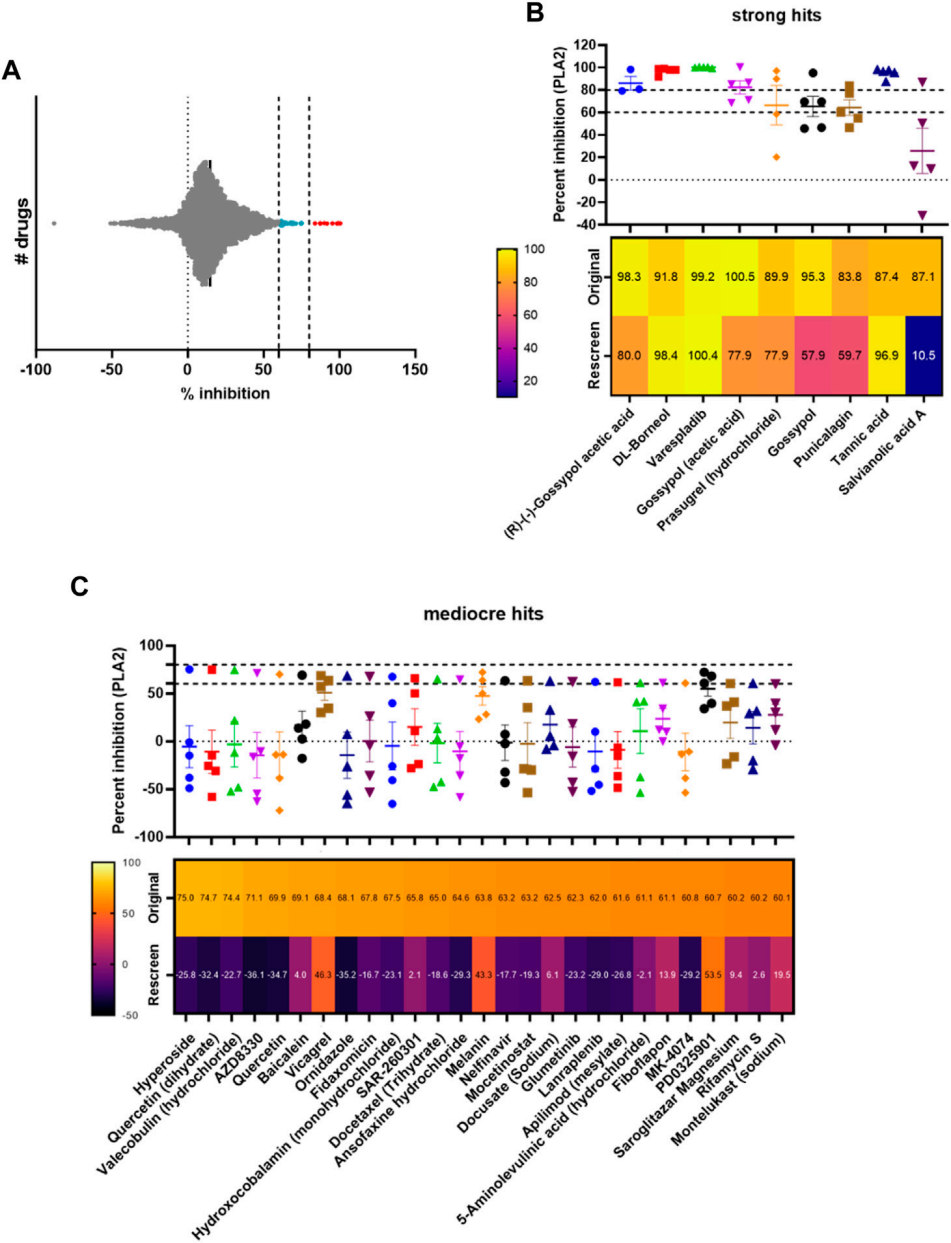
Screening of a commercial library and assessing hit reproducibility for strong and mediocre hits. **(A)** Visualisation of the distribution of hits, with strong hits highlighted in red (>80%) and mediocre hits in light blue (60% < x < 80%). **(B)** Percentage PLA_2_ inhibition for strong hits. The original data from the screen and rescreens are presented, and the median value (3 ≤ n ≤ 5) across all datasets is displayed with SDs. Below, a heat map displaying the values identified in the original screen versus the average values in the rescreen (2 ≤ n ≤ 4) is presented. **(C)** Percentage PLA_2_ inhibition for mediocre hits with SDs, with heat map below showing the original screen value versus the average value in the rescreen (bottom). Drug names are presented underneath each heat map.

Following method validation, we next wanted to test the ability of our assay to identify novel PLA_2_ inhibitors by screening a commercial drug library. To this end, we purchased a bespoke library sourced from MedChemExpress, which we screened against the medically important and PLA_2_-rich venom of *D. russelii* (Faisal et al., 2021; Laxme et al., 2021). The library was curated to contain diverse chemistry of 3,547 approved or post-phase I clinically trialled drugs (i.e., diversity of those that have previously completed preclinical and clinical studies for a wide range of diseases), as well as having well-characterised bioactivity, safety and bioavailability properties, making them amenable for drug repurposing. The drugs were plated at a final assay concentration of 10 µM in 384-well format with controls on either side of the plate. As our assay setup allows for a throughput of ∼2,800 drugs/day/venom, we were able to complete the screen for this library in under 2 days using single point measurements. The percentage inhibition of PLA_2_ activity was calculated relative to the positive (varespladib, set at 100%) and negative (DMSO, venom-only, 0%) controls and resulted in nine strong hits (percentage inhibition >80%, 0.25% hit rate) and 27 mediocre hits (percentage inhibition 60% < x < 80%, 0.76% hit rate). The majority of drugs are not significant inhibitors of PLA_2_ activity and fall within the −10% to 30% inhibition interval, with the data following a gaussian distribution as confirmed by the Shapiro-Wilks test for normally distributed data (W = 0.97, *p*-value = 2.13e−26) (Figure 3A).

We next selected all hits displaying >60% inhibition and independently rescreened them in the assay (Figures 3B, C). With one exception (salvianolic acid A), strong hits were consistently confirmed as PLA_2_ inhibitors in the subsequent independent runs (Figure 3B), with average inhibition values of at least 60% in eight of the nine hits, and at least 80% in five of the nine hits. Varespladib, which is also part of the MedChemExpress commercial drug library, was also detected as a hit with >99% inhibition of PLA_2_ activity. Conversely, mediocre hits were much more variable and, when retesting, the majority were revealed as false positives; only three of the 27 originally detected mediocre hits displayed average inhibition values of >40% upon rescreen (between 43% and 53% inhibition, Figure 3C). This suggests the need to implement a stringent cut-off—in this case the application of the “strong hit” threshold of 80% inhibition appears appropriate—and reconfirm hits before progressing any drugs into downstream analyses.

Of the strong hits recovered, gossypol, a phenolic compound derived from the cotton plant, appeared three times as an independent hit (mean inhibition between screen and rescreen of 89.2%, 89.2% and 76.6% for *R*-(-)-gossypol acetic acid, gossypol acetic acid and gossypol, respectively). Other plant compounds we recovered were punicalagin (mean inhibition of 71.7%), a compound found in pomegranates, tannic acid (92.1%), a polyphenol found in various woody plant species, and DL-borneol (95.1%), a terpene derivative from the teak family. Additionally, prasugrel hydrochloride, a platelet aggregation inhibitor, was also among our top hits (mean inhibition of 83.9%). We decided to pursue all strong hits with average inhibition values of >70%, including *R*-(-)-gossypol acetic acid, DL-Borneol, punicalagin, and prasugrel hydrochloride. Tannic acid was excluded from this selection as it is a likely false positive since it is known to react with the exposed -SH group present in the substrate and prevent the reaction between the latter and DTNB (Chen et al., 2022).

### PAINS filtering of drug hits

All compounds were virtually screened for substructures common to Pan-Assay INterference compoundS (PAINS) (Baell and Holloway, 2010). In our approach we screened the structures of all compounds for known PAINS substructures and flagged compounds containing these structures as being more likely to represent a false positive result. Out of all the strong and mediocre hits identified in the primary HTS, we identified 15 PAINS flagged structures (Supplementary Figure S3) of which four were strong hits. Gossypol, punicalagin, tannic acid and salvianolic acid were all flagged as PAINS positive due to containing catechol moieties—a moiety known to be have promiscuous reactivity, redox activity and to chelate metal ions (Matlock et al., 2018).

As previously mentioned, tannic acid was excluded due to documented reactivity with free thiols (Chen et al., 2022). Gossypol was also flagged due to containing the catechol moiety, but also for containing reactive aldehyde groups which have been documented to form Schiff bases with primary amines (Kovacic, 2023). This is supported by the identification of gossypol as a promiscuous compound using data-mining and deep-learning approaches (Yu et al., 1997; Bisson et al., 2016). Despite this limitation, owing to its documented inactivation of PLA_2_ in prior biochemical assays, we carried gossypol forward for further inhibitory profiling (B.-Z. Yu et al., 1997).

While lacking any reports of promiscuous behaviour in HTS, punicalagin, like tannic acid, is a naturally occurring tannin and contains many catechol moieties which may lead to false positives in our assay. This is supported by single-digit micromolar potency or less in 17 separate reported bioassays on the Chembl database (https://www.ebi.ac.uk/chembl/compound_report_card/CHEMBL416615/, accessed September 2023). Nevertheless, punicalagin was further shown via rescreening to inhibit PLA_2_ activity and was taken forward for further characterisation.

### Visualisation of the HTS dataset

To visualise the HTS chemical space and to identify trends among closely related scaffolds identified as PLA_2_ inhibitory molecules we employed Tree Manifold Approximation Projection (TMAP) (Probst and Reymond, 2020). TMAP visually represents chemical space as a tree-like manifold, grouping similar compounds into branches based on their proximity, revealing meaningful patterns and relationships within chemical space.

**FIGURE 4:**
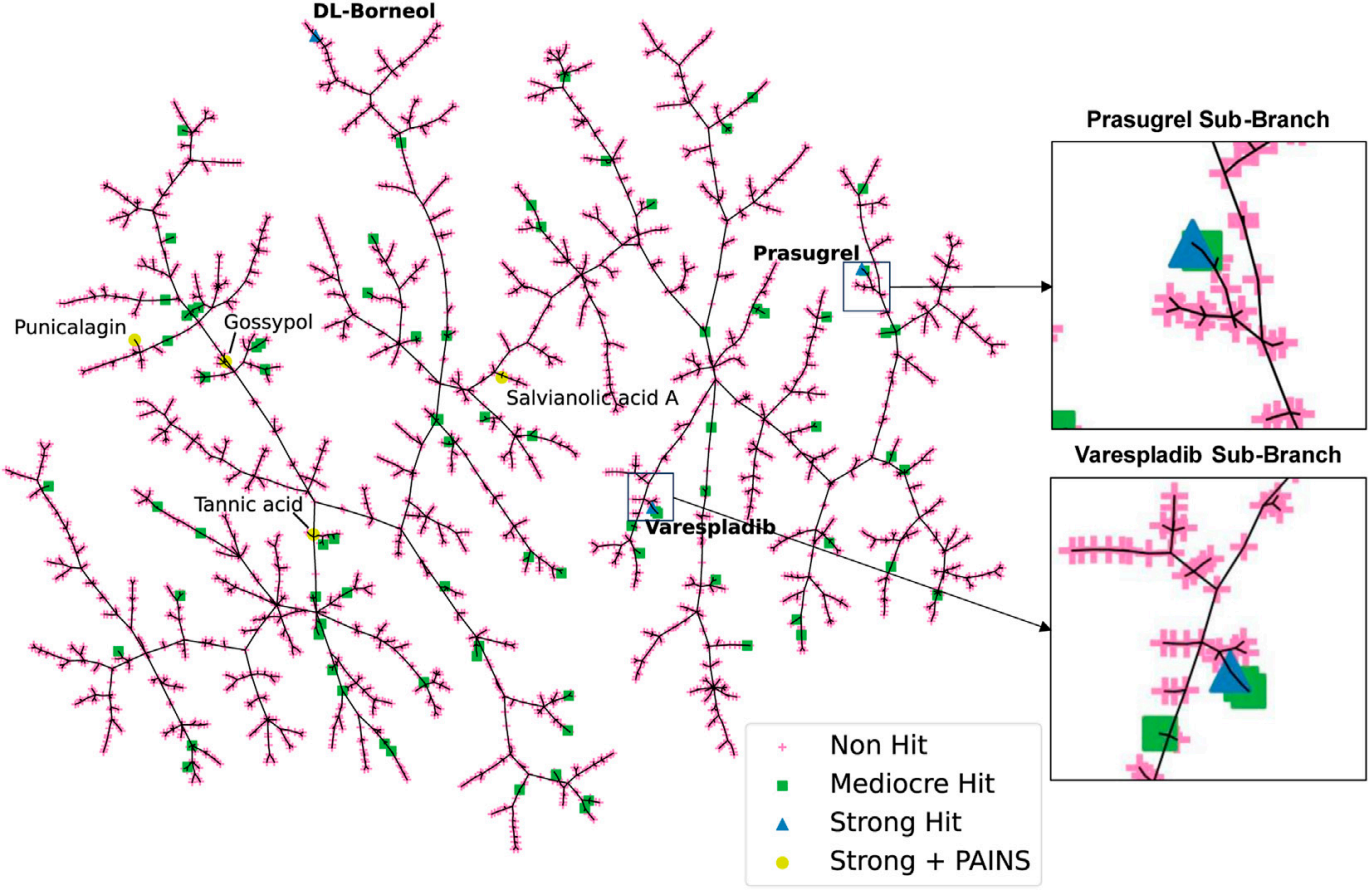
TMAP representation of the chemical space covered by our HTS library. Non-hits are shown as pink crosses, mediocre hits are shown as green squares, and strong hits are shown as blue triangles. Strong hits containing PAINS fragments are shown as yellow circles.

Visualisation of our dataset (Figure 4) shows that mediocre and strong hits are well distributed across the manifold with most mediocre hits isolated from other compounds. Most strong hits, including those flagged for containing PAINS related substructures, are similarly isolated on sub-branches, with the exception of varespladib and prasugrel, which share their sub-branches with mediocre hits fiboflapon and vicagrel, respectively. Fiboflapon is a potent inhibitor of 5-lipoxygenase-activating protein which shares a common *N*-aryl-2,3-disubstituted indole core with varespladib. Similarly, vicagrel, a closely related analogue of prasugrel, is an antiplatelet prodrug based on clopidogrel and which is converted sequentially by esterases and cytochrome P450 enzymes into an active metabolite common to both prasugrel and clopidogrel. The close proximity of these two strong hits to closely related moderate hits is suggestive of common inhibitory scaffolds.

### Exploring the breadth of inhibition and potency of selected strong hits

**FIGURE 5:**
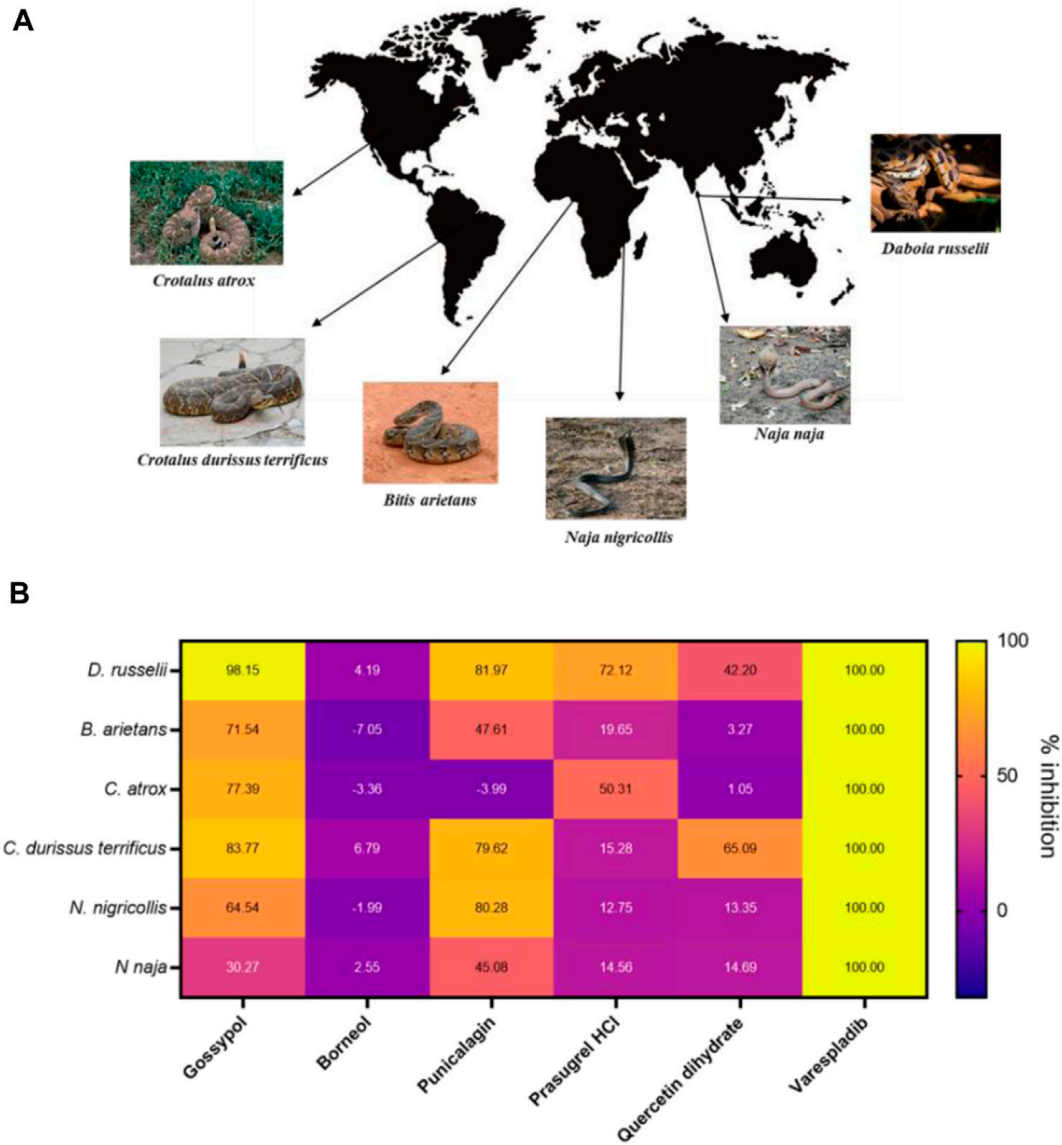
The PLA_2_ inhibitory capability of selected strong hits against different snake venoms. **(A)** Geographical distribution of a selection of PLA_2_-rich venoms spanning both viper and elapid snakes. Images either belong in the public domain or are displayed under a creative commons license; photographers Gary Stolz (*C. atrox*), Leandro Avelar (*C. durissus terrificus*), Kelly Abram (*B. arietans*), Lucy Keith-Diagne (*N. nigricollis*), Dr. Raju Kasambe (*N. naja*) and Tushar Mone (*D. russelli*). **(B)** Heat map displaying PLA_2_ inhibition values (in percentages) of selected drugs against those six venoms. Drugs were assayed at a top dose of 10 µM against an optimised venom amount which elicited a strong signal in the assay: *D. russelii* (30 ng), *N. naja* (40 ng), *B arietans* (40 ng), *C. atrox* (30 ng), *N. nigricollis* (5 ng) and *C. durissus terrificus* (12.5 ng). Varespladib was used as the positive control and was normalised as 100% inhibition.

As our original screen only assessed PLA_2_ inhibition for *D. russelii* venom, we wanted to understand whether our remaining hits were able to inhibit the PLA_2_ activity of a variety of PLA_2_-rich venoms at a top dose of 10 µM. As such, we chose venoms spanning a wide spectrum of geographical locations from Asia, Africa, South and North America, including both vipers (*Crotalus atrox* and *Crotalus durissus terrificus* from the *Crotalinae* subfamily and *Bitis arietans* from *Viperinae*) and elapids (*Naja naja* and *N. nigricollis*) (Figure 5A). This is important, because while both vipers and elapids have PLA_2_ toxins in their venoms, these evolved independently via the duplication of different genes encoding different PLA_2_ subclasses (IIA and IB, respectively) (Lynch, 2007; Fry et al., 2008). Following approved industry practices, we commercially sourced re-synthesised versions of our top hits to ensure their inhibitory activity was maintained independent of the material present in the commercial drug screening library. In addition to our selection of remaining strong hits, we also chose to test quercetin dihydrate, a compound which was previously shown to inhibit enzymatic PLA_2_s from *C. durissus terrificus* venom (Cotrim et al., 2011), and was initially recovered as a mediocre hit (inhibition value >70%) but failed to inhibit *D. russelii* venom in the rescreen (Figure 3C).

Gossypol, punicalagin and prasugrel hydrochloride all displayed PLA_2_ inhibition values above 72% against the venom of *D. russelii*, with gossypol and punicalagin exhibiting considerable breadth of inhibition against a selection of viper and elapid venoms (>70% inhibition against four venoms for gossypol and >80% inhibition against three venoms for punicalagin, Figure 5B). Interestingly, the new stock of quercetin dihydrate led to 42% inhibition of the PLA_2_ activity of *D. russelii* venom, and also partly inhibited the PLA_2_ activity of *C. durissus terrificus* venom (∼65%), in line with previous reports (Cotrim et al., 2011). However, the ineffectiveness of quercetin at inhibiting the PLA_2_ activities of the other four venoms tested (all <15% inhibition), coupled with its known promiscuity (Giannetti et al., 2008), likely limit its translational potential. Despite being a strong hit in both the primary and rescreen of the library, DL-borneol did not re-test as a hit against any of our six venoms, perhaps indicating issues with compound resynthesis. Contrastingly, the known PLA_2_ inhibitor varespladib (also our positive control) displayed the best cross-species effectiveness, with potent PLA_2_ inhibitory activity observed against all six diverse snake venoms, reducing readings to control levels. These findings highlight that this previously described drug remains the standout inhibitory molecule for inhibiting PLA_2_ venom toxins, despite the increased chemical space explored in this commercial drug library screen.

**FIGURE 6:**
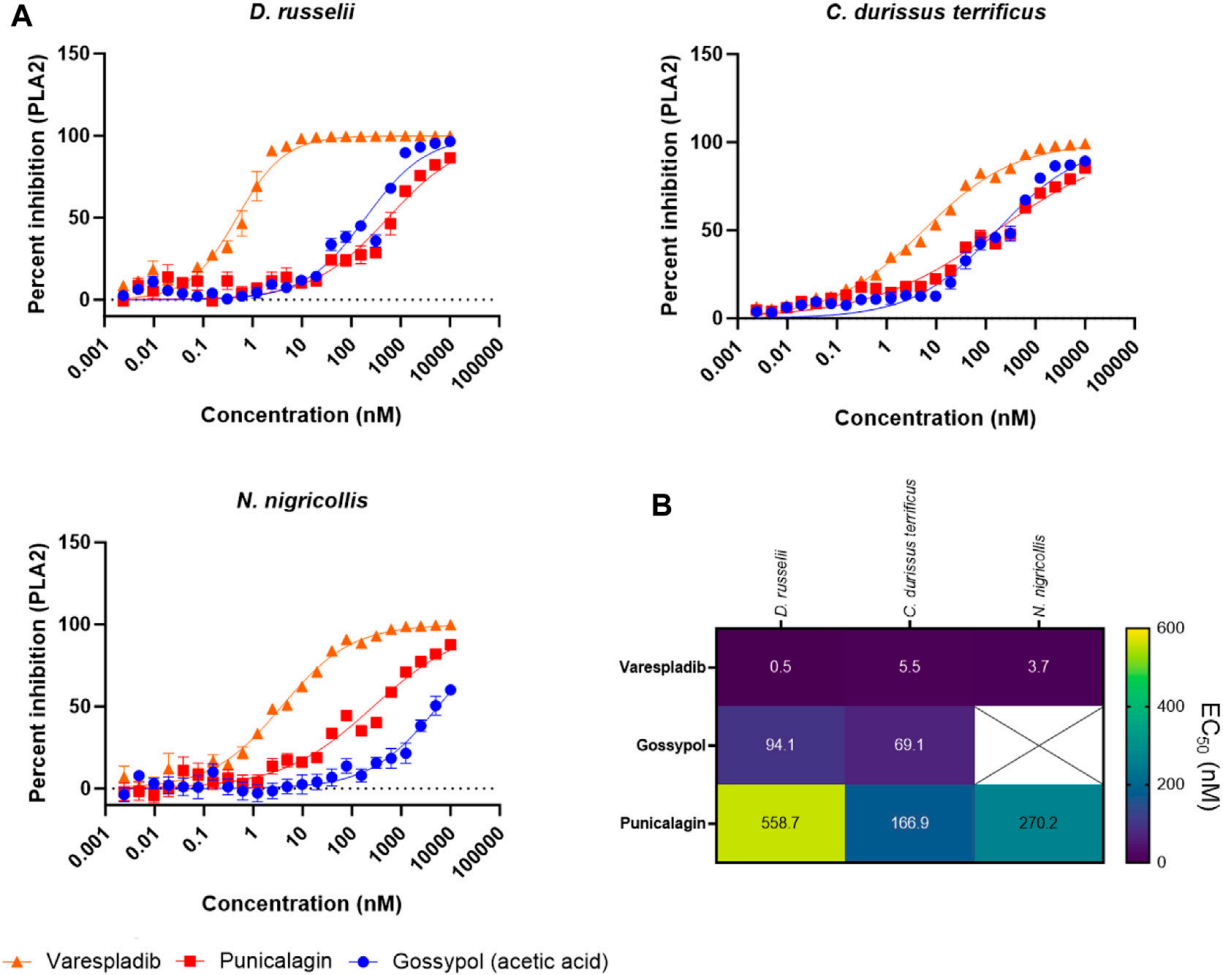
Testing the PLA_2_ inhibitory potency of the top three drug hits against diverse snake venoms. **(A)** EC_50_ curves of venom PLA_2_ inhibition for *R*-(-)-gossypol acetic acid, punicalagin and varespladib against three snake venoms (*D. russelii*, *C. durissus terrificus* and *N. nigricollis*). Curves were generated using 2-fold drug dilutions (*n* = 4), which spanned the 5 pM - 10 µM concentration range. **(B)**. EC_50_ values for the drugs against the three venoms. The crossed-out space denotes that the EC_50_ was not reported as the top inhibitory dose (10 µM) did not yield >80% inhibition of PLA_2_ activity.

To better explore the potency of our broad-spectrum hits, we next decided to generate EC_50_ curves for our three top performing drugs (varespladib, gossypol, and punicalagin) against three venoms. We chose the venoms of *D. russelii*, *C. durissus terrificus* and *N. nigricollis* because, with one exception (gossypol against *N. nigricollis* venom), all venoms were effectively inhibited by all three drugs at the top dose of 10 µM (Figure 5B, at least 71% inhibition). Our range of drug serial dilutions covered a concentration interval from 5 pM to 10 µM (Figure 6A). Varespladib was consistently the most potent PLA_2_ inhibitor with EC_50_s ranging from 500 pM against *D. russelii* venom to 5.5 nM against *C. durissus terrificus*. Gossypol was slightly more effective than punicalagin for the two viper venoms tested (Figure 6B) with EC_50_s in the low nanomolar range (94.1 and 69.1 nM for *D. russelii* and *C. durissus terrificus* respectively), but provided only partial inhibition for *N. nigricollis* venom at the top dose tested (10 µM). Similarly, punicalagin was most effective against the venom of *C. durissus terrificus* (EC_50_ of 166.9 nM), but its potency decreased to intermediate levels for the other two tested venoms (558.7 and 270.2 nM for *D. russelii* and *N. nigricollis* venoms respectively). Overall, these data demonstrate that the validated screening assay enables the identification of novel inhibitors that display strong to moderate inhibition against snake venom PLA_2_s and that our methodology is appropriate for broad-spectrum efficacy and EC_50_ testing.

## Discussion

Here we present the miniaturisation of a colorimetric assay which allowed us to assess PLA_2_ activity in snake venoms in a high throughput manner. We show that this methodology allows for a throughput of ∼2,800 drugs/day/venom and is therefore suitable for determining broad-spectrum inhibitory activities and potencies of novel PLA_2_ inhibitors of value for snakebite drug discovery. We have optimised the assay to ensure reproducibility within and between 384-well plates, and which resulted in Z′ values of >0.5. Using the commercially sourced MedChemExpress repurposed drug library we demonstrated the applicability of the assay for snakebite drug screening, and identified novel compounds capable of neutralising venom PLA_2_ activities. Our screening activities further contextualised varespladib as a potent and broad inhibitor of snake venom phospholipase toxins.

One important consideration for identifying hits was the stringency of our self-imposed cut-offs, with more stringent cut-offs ultimately required to ensure reproducibility given the relatively narrow assay window. As such, we defined strong hits as those exhibiting >80% PLA_2_ inhibition and these were validated in follow-up experiments to possess a true positive rate of 88.9% (8 out of 9). Contrastingly, false positives were abundant in the inhibitory interval initially assigned as mediocre hits (60% < x < 80%; 24 out of 27 initial mediocre hits; 88.9% FPR), leading us to largely exclude these from follow-up studies. One exception was quercetin, a compound retained because it was previously shown to inhibit the enzymatic PLA_2_ activity of *C. durissus terrificus* venom (Cotrim et al., 2011). Although we confirmed this activity against this same venom in our assay (Figure 5), this compound was not effective against other venoms, and thus appears not be a potent broad spectrum PLA_2_ inhibitor.

Among the nine strong hits identified from the primary screen, we noted an abundance of plant-derived compounds such as gossypol (isolated from cottonseed), punicalagin (a compound found in pomegranates), salvianolic acid A (from *Salvia miltiorrhiza* which is used in traditional Chinese medicine) and tannic acid (a polyphenol found in many plant species). Tannic acid likely represents a false positive as it is known to react with the exposed -SH group present in the assay substrate, thereby preventing its reaction with DTNB (Chen et al., 2022), and thus was excluded from follow up studies. Further, while salvianolic acid A failed to reproducibly inhibit *D. russelii* venom in the rescreen and was dropped from downstream experiments, both gossypol and punicalagin consistently displayed inhibition of at least >59% of the PLA_2_ activity of this venom. Gossypol was a hit in our library in three different instances (*R*-(-)-gossypol acetic acid, gossypol acetic acid and gossypol) and displayed inhibitory values between 95% and 100% against *D. russelii* venom (Figure 3B).

Gossypol is an orally active polyphenolic aldehyde which was trialled as a male contraceptive in the 1970s in China and shown to be efficacious and well tolerated at a dose of up to 20 mg/day (Liu et al., 1987; Coutinho et al., 2000). It is non-teratogenic (Beaudoin, 1985) and was shown to induce G0/G1 cell cycle arrest, inhibit DNA replication and lead to apoptosis (Zhang et al., 2003). In addition, gossypol inhibited proliferation in non-small cell lung carcinoma cells (Wang et al., 2018). While gossypol is flagged for PAINS associated substructures, and is documented as being a promiscuously reactive compound (Bisson et al., 2016; Matlock et al., 2018; Kovacic, 2023), we carried gossypol forward for further characterisation owing to reported inhibition of secreted PLA_2_ activity (B. Z. Yu et al., 1997). In our assay, gossypol showed broad-spectrum PLA_2_ inhibition across various viper and elapid venoms (Figure 5B), with at least 64% neutralisation of PLA_2_ activity across four other venoms when tested at 10 µM. However, gossypol appears to more effectively inhibit viper venom PLA_2_s (type IIA), with 71%–98% inhibition observed against the four viper species, compared with only 30% and 64% inhibition against the two elapid venoms (type IB PLA_2_) (Figure 5B). This was also the case based on calculated EC_50_s, which were in the nanomolar range for the two viper venoms (*C. durissus terrificus*, 69.1 nM; *D. russelii* at 94.1 nM), while an EC_50_ could not be precisely estimated for the elapid *N. nigricollis* due to a lack of complete inhibition at the highest dose tested.

One other plant-derived hit displaying >79% PLA_2_ inhibition across three snake venoms was punicalagin, one of the largest phenolic compounds known to date. Punicalagin was shown to possess antioxidant, anticancer and anti-inflammatory activity (Seeram et al., 2005; Danesi and Ferguson, 2017; Berdowska et al., 2021) while being non-toxic at high doses in rodents (Cerdá et al., 2003). Punicalagin was also flagged as containing PAINS related substructures in our virtual screen, however promiscuous biological activity has not been reported. While showing a more reduced spectrum of inhibition against the different snake venoms when compared to gossypol, punicalagin did display higher nanomolar EC_50_s against both viper and elapid venoms, with a value of 166.9 nM against *C. durissus terrificus* and 270.2 nM against *N. nigricollis* venom, though reduced potency was observed against *D. russelii* venom (558.7 nM) (Figure 6). In contrast to these plant-derived compounds, the platelet aggregator inhibitor prasugrel failed to provide more than mediocre inhibition against any of the other snake venoms tested (>50% inhibition observed for only two venoms). While prasugrel was found to possess poor broad-spectrum activity, visualisation of HTS chemical space brought the closely related platelet inhibitor vicagrel as a mediocre hit—this suggests that their common scaffold may have inherent, albeit weak, PLA_2_ inhibitory activities.

Despite the application of the optimised PLA_2_ inhibition assay for the discovery of novel venom toxin inhibitors via a high throughput screening approach, none of the hits identified exhibited equivalent or superior PLA_2_ inhibitory potencies to that of varespladib (potent inhibition against the six diverse venoms at 10 µM and EC_50_s in the 0.5–5.5 nM range across all three venoms tested) (Figures 5, 6), which represents the current “gold standard” PLA_2_ inhibitor in snake venom research (Lewin et al., 2016; 2022; Clare et al., 2021). Moreover, in addition to the limitations associated with reduced potency and inhibitory breadth, the phenolic compounds identified in our screen are relatively large molecules possessing several structural characteristics (Supplementary Figure S3; Supplementary Table S1), which render them suboptimal for progression for drug development purposes.

To be considered for lead development, a compound requires suitable physicochemical properties in addition to specific target affinity. Beyond the rule of five (Lipinski, 2000), ligand efficiency (LE) and ligand lipophilicity efficiency (LLE) are two metrics in modern early drug discovery that predict the affinity and quality of screened hits against a target (Hevener et al., 2018). The LE metric calculates ligand affinity using activity values (percentage inhibition values in this case) and the number of heavy atoms (Hopkins et al., 2004) and as such the values depend on their molecular weight. On the other hand, LLE accounts for the lipophilic properties of the molecules (LogP). Candidate drugs possessing higher LE and LLE metrics should be preferred during target-based drug discovery programs when compared to active ligands against that target (Leeson and Springthorpe, 2007). In addition, Gleeson (Gleeson, 2008), suggested that along with good activity profiles, lower molecular weight and LogP values must be emphasized in hit compounds for improved ADMET properties. Amongst our hits, LE values decreased with an increase in molecular weight/heavy atoms. When compared to varespladib, gossypol and punicalagin both displayed decreased LE metrics (Supplementary Table S1) due to their larger heavy atom count. In addition, while salvianolic acid and punicalagin presented similar LLE metrics to varespladib (accounted by their lower logP approximations), they had some of the lowest LE metrics among our strong hits. Together, these characteristics make gossypol and punicalagin suboptimal molecules for further progression.

Although varespladib retains exciting promise pending forthcoming snakebite clinical trial outcomes (Lewin et al., 2022), the high attrition rate of drug development provides a strong rationale to continue the search for novel PLA_2_ inhibiting compounds. To that end, we are currently utilising the methodology described herein to explore the wider chemical space covered by several other drug libraries. We believe this approach will be useful for identifying novel repurposed drugs in the field of snakebite envenoming, as well as allowing for the initiation of medicinal chemistry campaigns to rationally optimise hits by improving DMPK characteristics amenable to snakebite indication.

Despite our success in miniaturising this assay and markedly reducing cost, we are aware that research kits for determining PLA_2_ activity remain scarce and expensive, especially for researchers based in LMIC where snakebite is prevalent. This could be addressed by companies either producing cost-effective detection kits in line with demand as the use of these kits expands, or by exploring the use of alternative, more affordable, PLA_2_ substrates, which could then be incorporated into newly validated laboratory methodologies alongside use of appropriate home-made buffers.

In conclusion, we have successfully miniaturised a commercial PLA_2_ assay for high throughput screening and validated it for snakebite drug discovery purposes using snake venom. We show that the method is robust and applicable for screening of PLA_2_ inhibitors across various distinct snake venoms, but that it also has the potential for wider applications, including exploring the inhibitory potential of other therapeutic molecules for snakebite (i.e., monoclonal antibodies) and those outside this specific field (i.e., in the context of other venoms, and/or human or bacterial phospholipases). In addition, the HTS approach and platform have also been successfully used in our lab to screen for inhibitors of other toxin families (SVMPs, Clare et al., 2024). Overall, we hope that this will prove to be a valuable approach that will enable the development of improved therapeutics for treating the world’s tropical snakebite victims.

## Data Availability

The raw data supporting the conclusion of this article will be made available by the authors, without undue reservation.
