# Supplementary material for: Optimizing drug discovery for snakebite envenoming via a high-throughput phospholipase A2 screening platform
Source: Front Pharmacol. 2024 Jan 11;14:1331224. doi: 10.3389/fphar.2023.1331224 (PMC10808766; doi:10.3389/fphar.2023.1331224)
Supplement: Supplementary file 1 [file DataSheet1.docx]

**Fig. S1. Optimising venom doses and substrate concentrations in the context of PLA_2_ activity.** Dilutions of *D. russelii* (DRUS) and *N. nigricollis* (NN) venoms were run using the 1.66 mM substrate stock solution recommended in the kit and a lower 1.33 mM substrate solution (D - decreased). The activity of the bee venom positive control is shown on the left under both conditions. Venom amounts from 2 µg down to 200 pg were tested in triplicate using a 10-fold dilution. The top two doses tested were too high and consumed the substrate instantly resulting in complete saturation of the signal and as such are not plotted as we could not calculate an accurate slope. Venom amounts were then chosen for screening such that the substrate concentration does not become limiting. These were 20 ng venom for DRUS and 5 ng of venom for NN (see also Figure 1B).


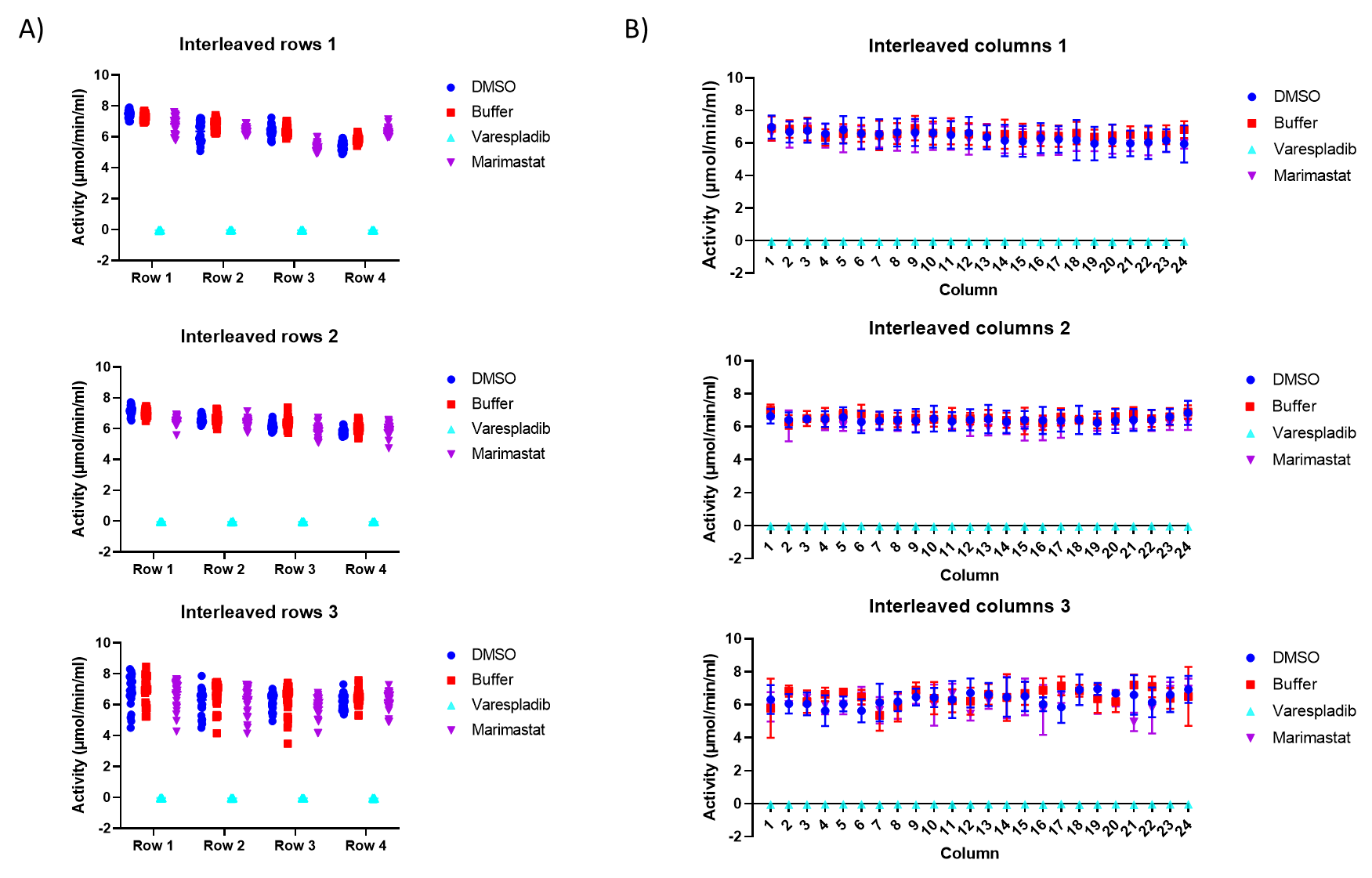


**Fig. S2. The data underpinning the quality control analyses generated for each of the interleaved plates.** All data included for the QC analysis. Interleaved data by row (A) shows any signal variability observed across the same condition (i.e. every 4^th^ row of the plate) and any change in activity noted from the top towards the bottom of the plate. (B) Interleaved data by column accounts for any drift observed from left to right across the 384-well plate. The data is presented as the mean and SD in each column.


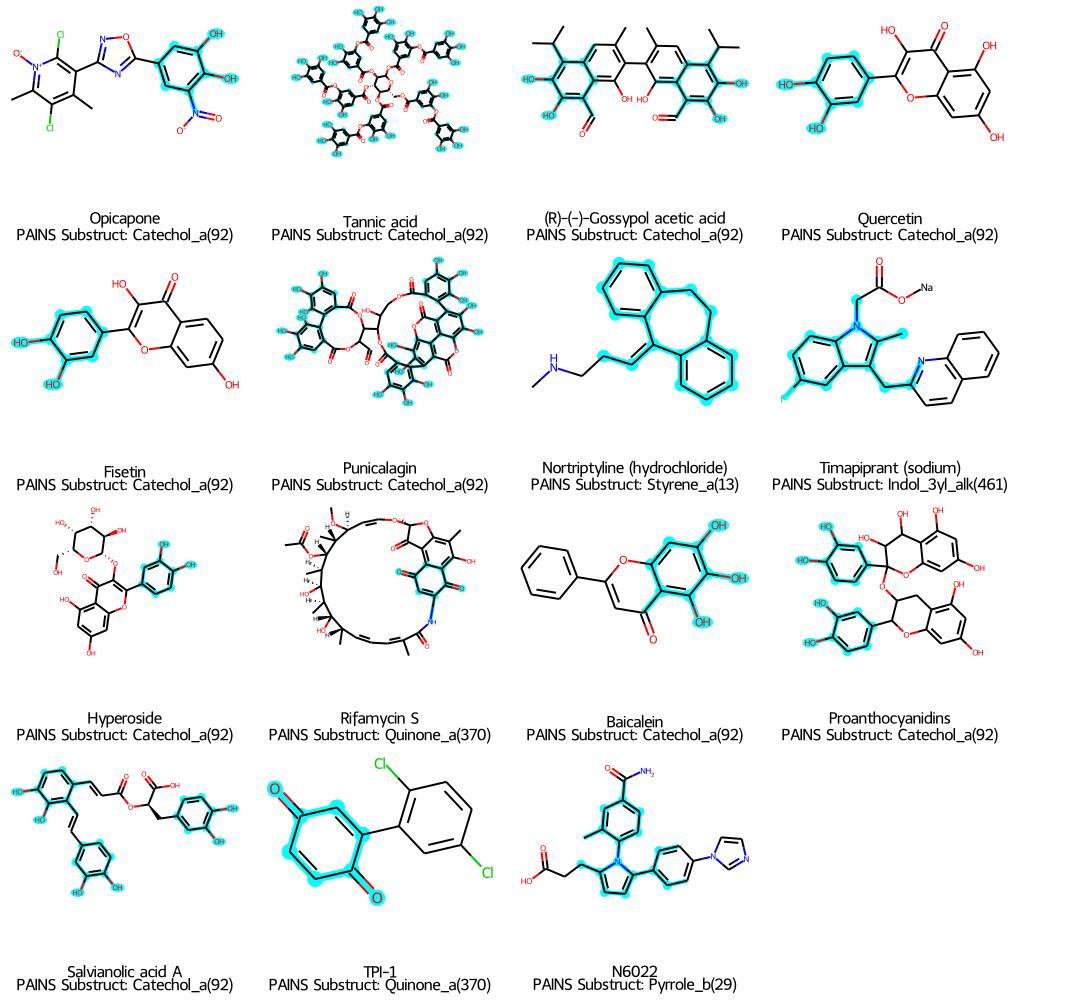


**Fig. S3. Structures found to be at least mediocre hits containing PAINS associated substructures.** PAINS substructure details the substructure flagged in the molecule, also shown highlighted in cyan.

**Table S1. In-silico approximations of ligand efficiency metrics for the strong hits.**

| Strong Hits | Percentage of Inhibition | Ligand Efficiency (LE) | Ligand Lipophilic Efficiency (LLE) | Molecular Weight | Heavy atom count | StarDrop™ approximation of LogP |
| --- | --- | --- | --- | --- | --- | --- |
|   Varespladib | 100.46 | 0.34 | 5.73 | 380.39 | 28 | 2.76 |
|   DL borneol | 99.24 | 0.87 | 4.96 | 154.25 | 11 | 2.81 |
|   Tannic acid | 98.3 | 0.07 | 1.47 | 1701.2 | 122 | 2.11 |
|   Gossypol acetic acid | 95.28 | 0.25 | 0.46 | 578.61 | 38 | 2.91 |
|   *R*-(-)-Gossypol Acetic Acid | 91.75 | 0.25 | 0.48 | 579.61 | 38 | 2.91 |
|   Prasugrel hydrochloride | 89.92 | 0.37 | 4.09 | 409.90 | 26 | 3.21 |
|   Gossypol | 87.39 | 0.25 | 0.49 | 518.55 | 38 | 2.91 |
|   Salvianolic acid A | 83.82 | 0.12 | 5.59 | 494.45 | 36 | 2.25 |
|   Punicalagin | 83.82 | 0.12 | 5.59 | 1084.7 | 78 | 0.72 |
